# Impact of Polyphenolic-Food on Longevity: An Elixir of Life. An Overview

**DOI:** 10.3390/antiox10040507

**Published:** 2021-03-24

**Authors:** Rosaria Meccariello, Stefania D’Angelo

**Affiliations:** Department of Movement Sciences and Wellbeing, University of Naples Parthenope, 80133 Naples, Italy; rosaria.meccariello@uniparthenope.it

**Keywords:** polyphenols, aging, blue zone, longevity, diet, antioxidant, oxidative stress, ROS

## Abstract

Aging and, particularly, the onset of age-related diseases are associated with tissue dysfunction and macromolecular damage, some of which can be attributed to accumulation of oxidative damage. Recently, growing interest has emerged on the beneficial effects of plant-based diets for the prevention of chronic diseases including obesity, diabetes, and cardiovascular disease. Several studies collectively suggests that the intake of polyphenols and their major food sources may exert beneficial effects on improving insulin resistance and related diabetes risk factors, such as inflammation and oxidative stress. They are the most abundant antioxidants in the diet, and their intake has been associated with a reduced aging in humans. Polyphenolic intake has been shown to be effective at ameliorating several age-related phenotypes, including oxidative stress, inflammation, impaired proteostasis, and cellular senescence, both in vitro and in vivo. In this paper, effects of these phytochemicals (either pure forms or polyphenolic-food) are reviewed and summarized according to affected cellular signaling pathways. Finally, the effectiveness of the anti-aging preventive action of nutritional interventions based on diets rich in polyphenolic food, such as the diets of the Blue zones, are discussed.

## 1. Introduction

Aging is a highly complex process marked by succeeding events that promote modifications in the normal functioning of an individual organism over time [1,2]. Numerous factors are involved in the occurrence of aging, comprising, epigenetic modifications, genomic instability, deregulated nutrient-sensing, loss of proteostasis, changed intercellular communication, telomere shortening, and cellular senescence [3]. Internal aspects include the regular biological activity of the cell, whereas the exterior influences implicate continuing sun-exposure, dietary deficiencies, hormonal difference, and other influences such as pollution and smoking [4,5]. Several theories have been hypothesized to define the aging phenomenon [6]. Denham Harman, in 1950, defined that aging is the effect of a significant synthesis of free radicals [7]. Free radical is a molecule or an atom with unpaired electrons, that owns the capability to make electronic couples. Free radicals are commonly synthesized during the metabolic reactions under physiological situations [8], but their production also takes place during contact to ultraviolet (UV) rays, cigarette smoke, and venomous molecules, as well as during emotional stress [9]. 

While accurate function of an organism needs metabolic reorganization of numerous chemical building blocks, there is also a damaging outcome that consequence from the accumulated byproducts of those reactions. The highly reactive molecules, created during oxidative metabolism, such as reactive oxygen species (ROS), have the capability to quickly oxidize, and thus injure several molecules. ROS, as well as hydroxyl/peroxyl radicals and peroxides, are formed through the regular processes of metabolism, e.g., oxidative phosphorylation and ATP synthesis. They can also have a helpful role; in fact they can defend the body from opportunistic pathogens, and provoke the production of hormones related to functioning communication between cells [10]. Disturbed ROS homeostasis, indicated as oxidative stress, is observed with crescent biological age [11], and it can consequence either from augmented ROS production or reduced capacity to remove ROS [12,13].

The oxidative stress contributes to senescence at the cellular level, and oxidative damage to diverse biomolecules takes place over time. ROS have been discovered to significantly contribute to age-related damage at the subcellular level through the destruction of numerous organic molecules including carbohydrates, proteins, DNA, and lipids [14,15]. Oxidative stress has been detected during aging [11], under certain pathological situations [16,17,18,19], as an effect of contractile action [20,21]. Moreover, oxidative stress is often aggravated by a diversity of environmental insults comprising metabolic processing of ingested food, contact with environmental poisons, and infection [22].

Research carried out in previous years shows that aging is much more malleable than previously thought. Aging is no longer as intractable and mysterious a process, offering new prospects for contributions from other branches of the physiological sciences [23]. The identification of cellular and molecular hallmarks of aging highlighted the probability for lifestyle-behavioral, comprising nutrition, to improve health span in humans [24].

In recent decades, the connection between aging and nutrition has been expansively studied in both humans and animals. Numerous food supplements that show antioxidant probability prevent and treat chronic conditions linked to ROS, which results in a healthier and longer life. Scientists have proposed that antioxidants have auspicious properties on both age-related and chronic syndromes, principally cancer and neurodegenerative syndromes [25].

Natural supplements have antagonistic actions against the body’s inflammatory and degenerative processes and have favorable consequence on the digestive and immune systems, thus ameliorating the quality of life [5,26]. Nutraceuticals are natural dietary molecules with medicinal capacities; in fact, the term “Nutraceuticals” is derived from “nutrition” and “pharmaceuticals” [27]. Agreeing for the foundation for novelty in medicine definition, nutraceuticals are “foods and food products” that have therapeutic importance and offer health positive effects, principally in the prevention and cure of age-related syndromes [27,28]. These molecules comprise alimentary supplements, functional foods, and herbal extracts, for example phytochemicals as polyphenols, which provide long-term health benefits [29,30].

This study argues the impact of a diet with anti-aging ability and particularly the consumption foods rich in polyphenols.

## 2. Polyphenols: Anti-Senescence Nutraceuticals

Numerous natural products/nutraceuticals derived from food, plants, and other organisms have been evaluated for their beneficial effects for health.

Polyphenols, organic compounds found copiously in plants, have become an incipient field of interest in nutrition in latest decades. A growing body of research shows that polyphenol consumption may play a vital part in health through the setting of metabolism, weight, chronic syndromes, and cell proliferation, and minor risks of chronic and age-related degenerative syndromes. Animal, human, and epidemiologic searches demonstrate that several polyphenols have antioxidant and anti-inflammatory capabilities that could have preventive and/or therapeutic effects for non-communicable diseases, such as cardiovascular disease, neurodegenerative syndromes, cancer, and obesity [31,32,33].

**Figure 1:**
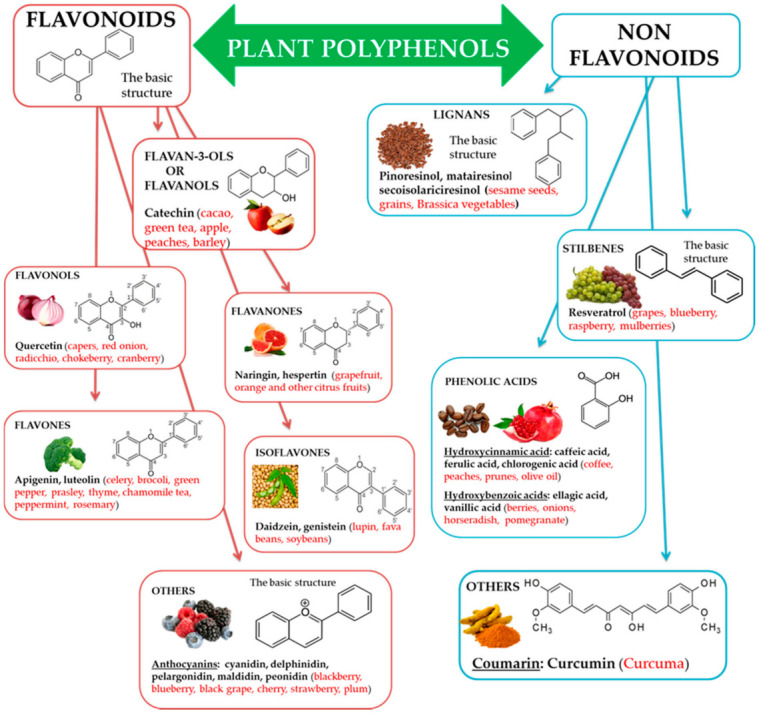
Polyphenols, subclasses, basic chemical structures, and representative polyphenolic-food sources (in red).

Natural biophenols are a wide group of phytochemicals (over 8000 described so far) found only in the plant kingdom; they are synthesized as secondary metabolites by the plant for defense against the attack by fungi, bacteria, and insects (phytoalexins). They are essential for a diversity of functions in plants, and they are accountable for organoleptic (flavor, color, astringency) and nutritional properties of plant-derived foods [34,35]. Chemically phenolic molecules consist of aromatic ring to which one or more OH− substituents are attached [36]. Despite of their chemical variety, the phenolic complexes are mainly separated into two subgroups: (1) flavonoids and (2) non-flavonoids. The first one is comprised of heterocyclic oxygen which are bonded with two aromatic rings and depends on the quantity of hydrogenation. They can be further divided into six subgroups, i.e., flavanols, flavanones, flavonols, isoflavones, flavones, and other, for example anthocyanins. Meanwhile, the second one contains aromatic rings which are attached to organic acids, like cinnamic and benzoic compounds. Lignans, tannins, stilbenes, and coumarin are also the subgroups of non-flavonoid molecules [37,38] (Figure 1).

Polyphenolic compounds have fascinated scientists internationally for their peculiar activities, such as anti-inflammatory, antioxidant power, and anti-carcinogenic properties [39]. Polyphenolic compounds have been acknowledged for a long time to nutritionists and the numerous scientists, and they are reputed the most potent natural antioxidants [40]. Since ancient times, humans ingest enormous amounts of polyphenolic compounds within vegetable source. These phytochemicals are present in abundance among fresh fruit and vegetables, especially leafy vegetables with a dark green color and in fruit with shades inclining to red (*Acai berries*), in cocoa, tea, and wine. Polyphenol-rich dietary foods are fruits (grapes, apples, berries, pears, and cherries), cereals, tea, red wine, dry beans, coffee, and chocolate, and can behave as active antioxidants [41,42]. Several fruits are very abundant in polyphenolic molecules, which are responsible for their taste, aroma, and color [43]. Apples, blueberries, grapes, raspberries, blackberries, plums, and strawberries are the most abundant in polyphenolic compounds [31,44]. Anthocyanins are the most frequently occurring polyphenolic compounds in fruit (especially abundant in colored fruit); then hydroxybenzoic and hydroxycinnamic and acids along with their products, tannins, flavonols, and catechins [45,46].

Several natural polyphenols studied for their healthy abilities are curcumin, detected in the tuber of *Curcuma longa Linn* (family Zingiberaceae) and an element of the curry; epigallocathechins, markedly epigallocatechin-3-gallate (EGCG), the flavanol discovered in green tea; quercetin and myricetin, flavonols found in tea, onions, cocoa, red wine, and in *Ginkgo biloba*. Other polyphenolic compounds have also been examined, with different results; these comprise tannic, ferulic, ellagic, caffeic acid, rutin, kaempferol, apigenin, fisetin, baicalein, luteolin, piceatannol, rottlerin, silibinin, and malvidin [47].

Most of the natural polyphenols are pigments, typically yellow, red, or purple, and can absorb UV radiation. This ability of natural polyphenols to act as sunscreens can reduce inflammation, oxidative stress, and DNA damaging effects of UV radiation in the skin [48,49]. Marine algae-derived polyphenols have been investigated for their photo protective activities. Phlorotannins, as dieckol, phloroglucinol, fucofuroeckol-A, and triphlorethol-A, isolated from marine brown algae, exhibited prominent protective effect against photo damage, induced by UVB radiation [50,51]. Thring et al. determined anti-collagenase, anti-elastase, and antioxidant activities of 21 plant extracts and correlated them with the total phenolic content. The white tea extract showed the highest inhibitory activity against enzymes as well as the highest antioxidant activity and phenolic content [52]. Strawberry extract containing mainly flavonoids and anthocyanins, protected dermal fibroblasts from oxidative stress induced through H_2_O_2_ [53]. Studies suggest that polyphenolic extracts can be useful ingredients for both sunscreens and after sun cosmetic products.

In recent decades, special attention has been paid to the anti-proliferative [54,55] or anti-oxidative effect of phenolic compounds [56,57,58,59,60,61,62] with suggestion supporting the probable involvement of polyphenols in the inhibition of various diseases [63,64,65,66,67,68].

Flavonoids are the main antioxidants in the food, and are recognized to preserve against cardiovascular diseases by reducing the oxidation of low-density lipoproteins. Luteolin, apigenin, chrysin, quercetin, datiscetin, morin, myricetin, and kaempferol are some of the most commonly found flavonoids [69]. Numerous studies, both in vitro and in vivo, have revealed that polyphenols have a brilliant capacity to interfere with our cellular signals, and stimulate a bio-regenerating response. This allows the production of other endogenous antioxidants: oxidative stress advances and this turns into an anti-inflammatory and anti-radical actions [32].

The properties of polyphenols are also being assessed in terms of communications with the gut microbiota [70]. Food components are characterized by a two-way communication with microbiota: (i) they can directly control their conformation and (ii) they are catabolized by the intestinal microbes to release metabolites that are more efficient and more easily absorbed than the native molecules [71]. It is valued that only 5–10% of the total polyphenols intake is absorbed in the small intestine and that 90–95% accumulates in the large intestine, where they undergo enzymatic alteration by the gut microbiota [72,73,74]. Since accumulative evidence supports the hypothesis that the gut microbiota are involved in the progress of human syndromes such as obesity, diabetes, metabolic syndrome, cancer, cardiovascular syndrome, and neurodegenerative diseases, it is conceivable that the defense against age-related syndrome development and progression, hypothesized for some anti-senescence mixtures, is related to the properties of such molecules on the microbiota [75]. In turn, the gut microbiota can prompt epigenetic variations, as validated in DNA methylation and histone variation of immune system cells.

The gastrointestinal microbiota of healthy human adults involves primarily bacteria belonging to the phyla *Firmicutes* and *Bacteroidetes* and, to a lesser extent, to *Actinobacteria* and *Proteobacteria* [71]. Inflammation may product in a higher level of aerobiosis and making of ROS, which deactivate the strictly anaerobic *Firmicutes* and encourage blooms of facultative aerobes, commonly named “pathobionts,” a condition that is habitually observed in the elderly [76]. The capacity of particular flavonoids, like quercetin, resveratrol, and catechin, to control the gut microbiota has been known in animal models [77]. Interestingly, apples, which are rich in flavonoids, have been related to a reduction in certain inflammation markers and variations in the gut microbiota of healthy mice [78].

## 3. Action of Polyphenols on Some Hallmarks of Aging

Natural polyphenols have been identified as essential plant compounds with anti-aging properties, such as blueberry polyphenols [79], black tea theaflavins [80], and procyanidins from apples [81], resveratrol [82], curcumin [83], and epigallocatechin gallate [84]. Current studies have revealed that polyphenols may modulate a number of phenomena that play a central role in the aging process. These phytochemicals possess a various and interesting pharmacological profile marked by connections with a broad range of biological targets.

Polyphenolic complexes have been revealed to modulate the redox status of cells, to alter cellular signaling, and to help avert the accumulation of injury in long-lived biological molecules such as nucleic acids, lipids, and proteins. This is accomplished both directly, through scavenging of reactive oxygen species, and secondarily, via interaction with transcription factors which coordinate the antioxidant reply. In fact, polyphenols have been revealed to prompt the overexpression of antioxidant enzymes such as superoxide dismutase and catalase [85]. Polyphenol-rich diets are strong antioxidants that function in vitro and in vivo. Polyphenol compounds such as resveratrol, quercetin, and curcumin have a defensive role against oxidative stress injuries [86,87].

**Figure 2:**
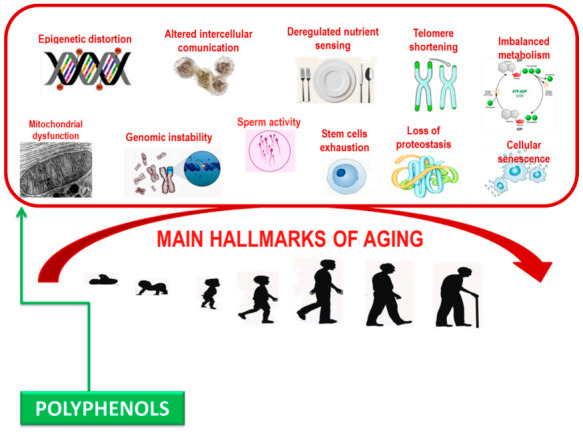
Main hallmarks contributing aging.

Polyphenols can damp down inflammatory signaling, modulate nutrient sensing pathways, and induce the selective apoptosis of senescent cells. Significantly, these biological processes become dysfunctional with age and are relevant in the pathogenesis of age-related syndrome [88,89] (Figure 2). Deciphering the accurate molecular mechanisms of capability of polyphenols in altering biological phenotypes of age-related syndrome is challenging due to the complexity of biological systems, where multiple diverse biochemical pathways can all contribute to a specific phenotypic outcome such as aging [90].

Over the past numerous decades, research in the biology of aging has attempted to discover “biomarkers” of aging. For example, telomere length has been a highly considered a biomarker of aging, as they shorten as individuals grow older. As proposed by Horvath’s efforts to use methylation markers as a biological clock [91,92], high-dimensional protein and/or metabolite profiles might emerge as ideal biomarkers of aging. Several studies suggest polyphenols as modulators of several of these aging indicators.

### 3.1. Polyphenols and Mitochondria

There is evidence that the accumulation of oxidative mitochondrial DNA damage during normal aging is a risk factor for the development of age-associated neurodegenerative disorders [93]. It has been revealed that the frequency of point mtDNA mutations augmented about 5-fold during an 80-year lifespan [94,95]. The efficiency of mitochondria in producing ATP significantly decreases when humans start aging thereby, allowing the increase of free radicals in these organelles as well as allowing the transit of free radicals through their membranes, in this way damaging other cellular elements [96]. These changes have facilitated to control the increase in oxidative stress and a reduction in energy production [11,97]. Senescence therefore encourages extensive metabolic and bioenergetics modifications [98]. The removal of dysfunctional mitochondria called mitophagy is critical for cell survival and health, particularly for neurons, as impairments might generally happen with aging [99,100].

In fact, two theories of aging regard telomere shortening and exactly mitochondrial DNA (mtDNA) variations and dysfunction. The modern evidence displays the presence of a strong linkage between these two theories suggesting common molecular mechanisms and a complicated telomere-mitochondria interplay during the humans’ aging [101].

As for the mitochondria, several information prove that polyphenols as resveratrol, curcumin, oleuropein, and hydroxytyrosol exert their positive abilities via the improved incentive of mitophagy intermediaries. The molecules stimulate the up-regulation of mitophagy and so increase the degradation of damaged mitochondria as well as the synthesis of new ones. Resveratrol encouraged peroxisome proliferator-activated receptor-γ coactivator-1α (PGC-1α) and mitochondrial transcription factor A (mtTFA) expression to augment mitochondrial biogenesis and stimulus proteins expression to control the balance of mitochondrial fission/fusion, thus preserving mitochondrial homeostasis [102,103].

Furthermore, all of the mentioned bioactive food molecules also own anti-oxidative abilities, which have a very important biological role in that since oxidative stress is deemed as one of the principal mediators of mitochondrial damage and decay in mitophagy occurring during aging [104]. The efficient elimination of not functional organelles and aggregated proteins is therefore basic to avert raised cellular stress and degeneration. The defensive action of antioxidants, ROS scavengers, and stimulators of mitophagy appears to be the focal mechanism of longevity and of reducing the risk for degenerative syndromes [99,101].

Additional of the most critical elements in aging is the accumulation of genetic damages throughout life. DNA solidity is compromised invariably by exogenous factors such as physical, biological, and chemical injuries. Further, DNA integrity is at risk by endogenous factors such as unrepaired defects in replication (point mutations, translocations, chromosomal gains, and losses), and telomere shortening. Oxidative DNA damage seems to be serious for aging, age-related syndromes and cancer. ROS and products of lipid peroxidation can have an effect on both genomic and mitochondrial DNA, leading to several types of DNA damage: double- and single-strand breaks, intra- and interstrand DNA crosslinks, DNA-adduct formation, DNA base and deoxyribose changes [105]. If the DNA mutations caused by free radicals are not corrected by particular repair mechanisms, these alterations remain even after numerous consecutive replication, transcription, and translation cycles [106].

### 3.2. Polyphenols and Telomeres

Shortening of telomeres is a well-known theory linked to aging. As a consequence of the problem of final replication, telomeres shrink in each generation of the cell until they reach a considerable length in the crisis phase of aging [107]. At this phase, cell division reduces speed noticeably, determining slow cell death. This phenomenon is called “replicative mortality.” The cells involved in growth, development, and reproduction (stem cells, eggs, and spermatozoa) synthesize large quantities of the enzyme telomerase, an enzyme involved in preserving the length of telomeric DNA [108]. Instead, most adult cells express little or none of this enzyme, producing these cells to age and ultimately die [101].

Levels of oxidative stress, antioxidants, mitochondrial alteration, inflammation, shortening of telomeres, and gene mutations all have a critical role in defining cellular aging. Studies propose that oxidative stress and the free radicals formed by it play an indispensable role in telomere shortening through reducing the action of telomerase or telomeric repeat-binding factor 2 (TRF-2) levels [87]. Shortening the telomere length harms health and leads to genomic variability and consequently jeopardizes the function of the cell cycle, and the cells enter the aging and apoptotic phases [87,109].

Nowadays, investigators are trying to increase telomerase activity and stabilize telomere length and prolong life by using antioxidant supplements such as polyphenols [110]. Studies and indication propose that polyphenols, with their antioxidant and anti-inflammatory capabilities, can affect telomere length and prevent shortening as far as possible. The antioxidant effects of diet on telomere function show that diet is a significant factor in determining telomere length status. A research indicated that leukocyte telomere length is considerably improved in subjects who take Mediterranean diet (MedDiet), rich in olive oil [111]. Proanthocyanidins and procyanidins are polyphenols found in grape seed extract. These polyphenols are powerful free radical scavengers, own anti-inflammatory capabilities decrease apoptosis, and impede hydrogen peroxide induced chromosomal injury in human lymphoblastic cells. Their free radical scavenging ability is 20 times more actual than vitamin E and 50 times more effective than vitamin C [112]. EGCG, and quercetin with strong antioxidant effect, may impede cardiac myocyte apoptosis by averting telomere shortening and TRF-2 loss [113]. Tea is rich in polyphenols, and other phytochemicals; a cross-sectional study of Chinese men and women found that elderly Chinese men had a helpful association with telomere length [114].

Therefore, studies propose that polyphenols, with their antioxidant and anti-inflammatory abilities, can affect telomere length and prevent shortening as far as probable and thus have powerful anti-aging capabilities [87].

Genetics is clearly significant in determining cellular aging in vitro and in vivo, and part of organismal aging may be dependent on cell division, with total cellular lifespan measured by the number of cell divisions (i.e., generations), not essentially by chronological time. This means that there is an intrinsic process occurring during cell growth which culminates in the interruption of cell division. If cellular age is controlled by a genetically determined counting programmer that controls the number of cell divisions, then it is central to define and understand the molecular pathways and regulation of this mechanism [109]. The studies have recognized the genes that can extend life expectancy and decrease age-related syndromes, including the Klotho gene. Polyphenols can influence intracellular function through activation of the Klotho gene, which induces the transcription factors, insulin-like growth factor 1 (IGF-1), and transforming growth factor (TGF-1β) [115].

### 3.3. Polyphenols and Sirt-1

Several reports highlighted that dietary supplementation of polyphenols may defend against neurodegenerative, cardiovascular, metabolic syndromes, inflammatory, and cancer by enhancing Sirt-1 deacetylase action. Sirtuins are a class of nutrient-sensitive epigenetic information regulators, including in promoting mammalian health, modulating cellular senescence and lifespan, and Sirt1 is called the longevity gene. Sirt1 is a (NAD^+^)-dependent deacetylase that targets a number of transcription factors, such as fork head box transcription factor (FOXO) 1, 3, and 4, p53, nuclear factor NF-κB, and peroxisome proliferator-activated receptor gamma co-activator 1 (PGC-1), moderating in turn a number of cellular stress adaptive responses. Sirt1 can deacetylase p53 in a NAD+-dependent manner to impede p53 transcription, modulating pathways involved in cellular and organismal aging [116]. Significantly, sirtuins are themselves controlled by diet and environmental stress [117,118,119]. Sirtuins impact multiple cellular pathways responsible for regulating gene expression, metabolism, DNA repair, apoptosis, and aging. Activating this pathway can increase life expectancy.

Resveratrol, curcumin, quercetin, tannins, and catechins may also be cited as molecules that increment sirtuins’ actions [120,121,122]. Resveratrol has been found to ameliorate brain health through numerous signaling pathways mechanisms through Sirt-1. The regulatory mechanisms include anti-inflammatory, anti-oxidative, anti-apoptotic processes and autophagy regulation, as well as increases in cerebral blood flow and enhancements in the plasticity of synaptic pathways [123]. Administration of polyphenols quercetin, silymarin, and naringeninin determined restorative actions on cognition and motor coordination in rats. These polyphenols inverted the age-induced insufficiencies in mono-aminergic neurotransmitters and amplified Sirt-1 levels and reduced NF-κB levels in hippocampus [124].

Chronic treatments with catechins, polyphenols present in many dietary foods, as gooseberries, apples, grape seeds, blueberries, strawberries, kiwi, red wine, green tea, cocoa, beer, cacao liquor, and chocolate, increase hippocampal Sirt-1 levels improving cognition in aged rats [125]. Therefore, polyphenols may defend Sirt-1 due to their antioxidant abilities and, in turn, moderate proteins affected by Sirt-1 action [122].

### 3.4. Polyphenols and Male Fertility

Aging has an important impact on male fertility, but the mechanisms diminishing fertility rate in elderly subjects are still poorly understood. The well-known endocrine route that sustains the progression of spermatogenesis and spermatozoa differentiation [126,127] declines due to steroid genesis defects. In addition, during the aging process morphological and functional alternations affect the testis, semen quality declines with changes in sperm morphology and concentration, and this causes defects in the acquisition of sperm motility [128]. At molecular level, sperm DNA damage, alteration in chromatin architecture mainly due to defective protamination, occurs. In parallel, the deregulation of epigenetic marks (i.e., non-coding RNA profile) in both spermatozoa and seminal plasma may affect the subsequent embryonic development and offspring health [129,130]. Oxidative stress, together with the decrease in antioxidant activity and mitochondria dysfunctions, is the main cause of testicular and sperm damage being ROS notably responsible for spermatogenesis failure, apoptotic loss of both germ and somatic cells, oxidative DNA damage, failure in gene expression and post-transcriptional gene regulation, APT depletion leading to insufficient axonemal phosphorylation in sperm tail, lipid peroxidation, and loss of sperm motility and viability, among the others [131,132].

Therefore, controlled antioxidant supplementation may be useful to preserve sperm quality along the life-span [132,133]. Furthermore, in animal models and humans antioxidants resulted to be useful to preserve semen quality before cryopreservation and after thawing [133] for in vitro fertilization (IVF) [134]. In this respect, several studies reported the effects of polyphenols supplementation on sperm quality parameters. For example, tea polyphenols, known as catechins, decreased the apoptosis rate of spermatogenic cells in rats with experimental varicocele [135]. Consistently, the ad libitum administration of 2% and 5% aqueous extract of green tea for 52 days increased sperm concentration and viability in rats, [136]. In the same study, the authors revealed spontaneous acrosome reaction and morphological changes in testis and epidydimis, with increased cauda epididymis epithelial height and decreased diameter and epithelial height of seminiferous tubules [136]. Similar effects on sperm vitality, motility, acrosome reaction, and morphological parameters in the seminiferous tubules and epididymis were observed following black tea administration [137]. Both green tea and black tea decreased serum levels of alanine transaminase and aspartate transaminase but black tea only increased creatinine levels [137]

Aqueous leaf extract of *Moringa oleifera*, the “miracle tree” containing a great number of bioactive compounds including polyphenols [138], reduced intracellular ROS production, DNA fragmentation and acrosome reaction without any effect on sperm motility, vitality, mitochondrial membrane potential and capacitation in human spermatozoa in vitro [139]. Quercetin improves the quality of cryopreserved human, dog and bull semen [140,141,142] and exerts protective effects against heavy metals induced oxidative injury in goat sperm and zygotes [143]. In vitro, resveratrol has protective effect on the sperm functions affecting motility, plasma zinc concentration, and acrosin activity in male infertility induced by body weight excess and obesity [144]. It also counteracts the detrimental effects of the polycyclic aromatic hydrocarbon benzo-α-pyrene on human sperm [145], and significantly improves the fertilization capacity in humans and animal models [146,147,148]. A comparative analysis of resveratrol and EGCG has been carried out on thawed boar spermatozoa revealing an increase in the total efficiency of fertilization, for both molecules [146]. Then, polyphenols may be useful to preserve spermatozoa quality parameter with potential application in IVF.

### 3.5. Polyphenols and Inflammation, Apoptosis, and Autophagy

Other essential changes in the aging process comprise chronic inflammation of the body’s cytokines (such as interleukin (IL)-6, tumor necrosis factor (TNF)-α). Anti-inflammatory action of polyphenols such as catechin, apigenin, luteoloside, ellagic acid, and rutin, has been detected in acute and chronic inflammation. The molecular mechanisms of polyphenols are associated with inhibiting nuclear factor kappa-light-chain-enhancer of activated B cells (NF-kB) pathways [149]. Resveratrol augmented insulin sensitivity, AMP-activated protein kinase (AMPK), peroxisome proliferator-activated receptor-c coactivator 1α (PPAR-co-1α) activity and anti-inflammatory microRNAs [150,151]. Polyphenolic compounds perform the anti-obesity action by inhibiting or decreasing lipid synthesis in adipocytes, moderating lipogenesis, decreasing inflammation and oxidative stress, and stimulating AMPK through stopping ATP synthesis [152].

Triggering programmed cell death is an effective mechanism to prevent cellular aging. In the process of apoptosis, older cells are destroyed and replaced with young cells. However, this protection against aging has many risks. During aging, inadequate cell death origins cancer to spread, and extreme cell death leads to tissue atrophy, connected with reduced life-span [153,154]. Polyphenols can constrain muscle atrophy and damage to the immune system from inhibiting the apoptosis process while enhancing this process is effective in clearing cancer cells. Nevertheless, there is a great deal of disagreement regarding the capability of these phytochemicals to promote or reverse apoptosis, and the relationship between apoptosis and the aging process needs further revisions and clarification.

Autophagy is an internal process that aids in lysosomal lack and removal of old and unwanted cell molecules, including proteins, lipid droplets, ribosomes, and other organelles, protecting cell homeostasis and survival under metabolic stress. Hence, autophagy protects the general health of the host, particularly in pathological situations such as cancer and diabetic cardiomyopathy. Numerous bioactive polyphenols, such as isoflavones and curcumin [155], are capable of causing autophagy. Optimal concentrations of EGCG were able to cause autophagy, anti-inflammatory effect [156], destroy lipid droplets in endothelial cells, and stimulate the degradation of endotoxins with anti-inflammatory effects [156]. Numerous studies suggest that the activation of autophagy by numerous polyphenols should contribute to their neuroprotective action. Tea polyphenols can activate autophagy by various mechanisms, such as the mammalian target of the rapamycin pathway (mTOR) [157]. Treatment with EGCG can cause autophagy, as it reduces the action of negative autophagy regulators that control apoptosis. In other words, EGCG is capable of amplifying autophagy, thereby slowing cell death mediated by apoptosis and thus increasing cell viability [158].

### 3.6. Polyphenols, Nrf2, and Proteostasis

One of the elegant mechanisms that cells have adapted to protect themselves against oxidative stress and other insults is Nrf2 pathway and the binding of this master transcriptional regulator with antioxidant response element (ARE) in the regulatory region of many genes, which leads to the expression of several enzymes with antioxidant and detoxification capacities. The nuclear factor E2-related factor 2 (Nrf2) and its invertebrate homologs have emerged as master regulators of cellular detoxification responses and redox status. These stress-sensing transcription factors function both in situations of acute challenge and as regulators of baseline antioxidant activity. The oxidative stress theory of aging posits that oxidative damage to biological macromolecules is a key driver of aging, and that, conversely, mechanisms that delay the accumulation of oxidation products in the cells and tissues of an organism can promote longevity. Consistent with this tenet and the established role of Nrf2 and its invertebrate homologs as master regulators of antioxidant gene expression, a number of studies support a function for the Nrf2 pathway in the regulation of lifespan. In both *C. elegans* and *D. melanogaster*, the genetic activation of the Nrf2 signaling can cause significant increases in longevity [159]. Under normoxic conditions, Nrf2 levels are low, predominantly due to binding to the negative regulator KEAP1 (Kelch-like ECH-associated protein 1), which facilitates Nrf2 ubiquitination and proteasomal degradation [160]. During increased oxidative stress, oxidative cysteine modification of KEAP1 alters its conformation, resulting in diminished binding to Nrf2. Nrf2, no longer subject to degradation, translocates to the nucleus where it binds to the ARE upstream of cytoprotective genes, e.g., NAD(P)H quinone oxidoreductase 1, glutathione S-transferase, and glutathione reductase [161], inducing their expression. The Keap1-Nrf2/ARE signaling pathway is an important defense system against exogenous and endogenous oxidative stress injury. These factors act to lower ROS and oxidative stress, while simultaneously reducing the cysteines in Keap1 and subsequently re-establishing baseline equilibrium of Nrf2 activity. Several studies have shown that polyphenols can induce Nrf2 function in different models [162,163]. For example, in endothelial cells resveratrol has anti-inflammatory effects that appear to be mediated by the induction of Nrf2 [159]; in mice, resveratrol improved renal function by activation of the Nrf2 and Sirt1 signaling pathways, ameliorating oxidative stress and mitochondrial dysfunction [162,164].

As well coordinating the antioxidant response, activation of Nrf2 has been demonstrated to increase proteasomal activity, allowing cells to control protein levels by regulated degradation. Activation of Nrf2 increases the expression and activity of the proteasome in a Nrf2-dependant manner [90,165].

Another molecular mechanism that prompts senescence is impaired protein homeostasis or proteostasis [166,167]. This is thought to be due at least in part to an increase in the accumulation of errors in translation, splicing, or molecular misreading, and to an increased production of oxidized and carbonylated proteins, and therefore systems are necessary which regulate and preserve a functional cellular protein pool. Proteostasis is a network of quality-control processes including protein clearance mechanisms that constrain the toxicity of misfolded proteins. Ensuring cellular protein homeostasis requires specific control of protein synthesis, folding, conformational maintenance, and degradation. A complex and adaptive proteostasis network coordinates these processes with molecular chaperones of diverse classes and their regulators functioning as major players. The most important systems for these removal processes are the “ubiquitin-proteasomal system” (UPS), the central proteolytic machinery of mammalian cells, mainly responsible for proteostasis, as well as the “autophagy-lysosomal system,” which mediates the turnover of organelles and large aggregates. Many age-related pathologies and the aging process itself are accompanied by a dysregulation of UPS, autophagy, and the cross-talk between both systems [166,167]. Failure to destroy the unfold proteins by proteostasis system will lead to the amassing and aggregation of these proteins and ultimately induce aging [168,169]. Although the protein quality-control networks ensure proteostasis under basal conditions, on conformational stress, such as increases in temperature or exposure to oxidative agents, many additional proteins become prone to misfolding, with proteins comprising the metastable sub-proteome being particularly vulnerable [166,170]

Irreversibly oxidized proteins must be degraded and replaced by de novo synthesized ones in order to maintain functionality and proteostasis of a cell. In the case a protein is oxidatively modified/damaged by ROS in an ongoing process, it undergoes a transition from slight functional decrease and increased solubility to a completely dysfunctional, unfolded, and insoluble structure that may be even resistant to mammalian proteases due to covalent cross-linking, depending on the amount of oxidative modification [167]. In order to counteract oxidative damage of cellular structures in redox-shifts, inflammation or oxidative stress, exceeding the “basic” amount of ROS produced in normal cellular function, there are powerful systems that can be induced, increasing the antioxidant capacity of the cell. If most important cellular ROS-scavenging enzymes are not sufficient to prevent a cellular redox shift, molecular redox-sensors such as Keap1/Nrf2 can be activated very quickly [167].

Polyphenolic compounds may enhance the efficacy of associated protein degradation by the proteasome and autophagy, and weaken oxidative stress [47,171]. Although these molecules act on numerous biochemical pathways, their activity in regulating the protein degradation mechanisms at different stages may be a suggestive therapy to stop the increase of misfolded proteins [172,173].

The ever-growing interest and public awareness surrounding the potential benefits of natural health products and polyphenols, in addition to their widespread availability and accessibility through nutritional supplements and fortified foods, has led to increased consumption. Foods can be fortified with polyphenols; dietary supplements that contain high doses of polyphenols can be developed. Some studies purport that excessive polyphenol consumption may have negative health effects in some sub-populations [174,175,176]. In such cases, toxicological testing may be required to ensure safe levels of intake. Then, too much may not be good and thus, dietary polyphenols may be beneficial in the correct amounts. The risk of consuming high doses of polyphenols from naturally polyphenol-rich foods is low. Therefore, the advice is to one should be content with eating a good diet for now and to take polyphenolic-food.

## 4. Examples of Polyphenolic-Food: Anti-Aging Super Food

Polyphenols are widespread constituents of vegetables, fruits, olive oil, chocolate, legumes, and beverages such as tea, wine, and coffee. The consumption of polyphenolic-food symbolizes a promising therapeutic approach to avert many chronic syndromes and ameliorate health. Main polyphenolic-foods comprise fruits (such as apples, peaches, pomegranates, plums, apricots, sweet cherries, etc.), berries (such as black elderberry, black chokeberry, plum, low bush blueberry, raspberry, blackcurrant, cherry, strawberry, blackberry, black grapes, prune, etc.), vegetables (such as globe artichokes, green chicory, red chicory, broccoli, red onion, spinach, curly endive, etc.), nuts (such as hazelnuts, chestnuts, walnuts, pecans, almonds, etc.), fruit juices (such as lemon juice, blood orange juice, etc.), soy (such as tofu, soy tempeh, soybean sprouts, soy yogurt, soy flour, etc.), green and black tea, red wine, cereals, coffee, and chocolate [38,41].

The consumption of berry-type fruits has become very common in current years because of their helpful properties on human health, comprising prevention of chronic syndrome, cardiovascular syndrome, and cancer. Regularly consumed berries, such as blueberries, strawberries, blackberries, red currants, white currants, blackcurrants, and raspberries, are a rich source of numerous polyphenols comprising quercetin, anthocyanins, and different types of phenolic acids.

**Blueberries** (656.00 mg/100 g) (according to the database Phenol-Explore) [177] have been labeled a “super fruit” for their capability to prevent or mitigate numerous syndromes, such as cardiovascular syndromes, diabetes, and cancer [178]. In recent years, blueberries have risen from relative obscurity to super food status following a quantity of published epidemiological researches, rodent trials, and human, that recommends blueberries may convey benefits to cognition and mood. Actions are seen following dose sizes easily achievable within a usual diet [32,179,180,181]. The high antioxidant potential of blueberry extracts has been linked with the improvement of aging symptoms [179]. Galli et al. have observed, in the hippocampus of rats, that supplementation with a cranberry extract may reverse the age-related decline of the heat shock protein [182]. In older rat models, blueberries have been observed to be active in ameliorating motor and cognitive comportment [183]. Furthermore, supplementing the diet with 5 mg/mL of cranberry extract significantly increased the lifespan of fruit flies by 10% [184]. Epidemiologically, intake of blueberries has been associated with slower rates of cognitive decline during the aging process [185]. Experimentally, daily supplementation with blueberry interventions has caused an enhanced neural action and enhanced working memory performance [186], and developed memory and executive function performance [187], in older adults. These properties are not limited to healthy aging. Blueberries have also revealed promise in age-related neuropathology. For example, daily blueberry supplementation improved memory performance [188], as well as neural action during a working memory exercise [189], in older adults formerly diagnosed with mild cognitive impairment.

**Strawberries** are fruits rich in anthocyanidins, such as cyanidin, pelargonidin, and proanthocyanidins (289.20 mg/100 g) [177]. Strawberries have antioxidant and anti-inflammatory capacities, capable of improving neuronal function and cognition [190]. In 245 subjects who developed AD in the mean follow-up of 6.7 years, increased consumption of strawberries resulted in a lower risk of Alzheimer’s dementia [191].

One of the food sources of polyphenols most frequently found in the diet is apples (*Malus domestica*) (250.89 mg/100 g) [177]. The apple is a fruit rich in phytochemicals; in particular they are abundant in polyphenols [192,193], such as rutin, chlorogenic acid, epicatechin, catechin, proanthocyanidin B2, and phloretin. The daily intake of apples is thought to reduce the incidence of cardiovascular syndromes and hypercholesterolemia [194]. The intake of apples can significantly reduce the danger of lung cancer, particularly in women. Numerous diverse researches have observed that the apple is active in slowing down the oxidation of low-density lipoproteins [195]. Apple polyphenolic compounds improved the lifespan of fruit flies by 10%. Furthermore, up-regulation of the CAT gene, SOD1, and SOD2 was detected [196]. Finally, concentrated apple juice showed a neuroprotective effect in normal aged mice and genetically compromised mice [5].

**Black rice** has been consumed for centuries in Asian countries such as China, Korea, and Japan. Black rice is considered as functional foods both its high content in γ-oryzanol, an ester mixture of ferulic acid and several kinds of phytosterols, and other bioactive molecules such as anthocyanins and other polyphenols [197,198,199,200]. Intake of 30 mg/dL of anthocyanins from black rice extended the lifespan of 14% of fruit flies [201]. In a research conducted on a mouse model with sub-acute aging, Huang et al. evaluated the action of black rice anthocyanins and found that black rice anthocyanins exhibit anti-aging, anti-fatigue, and anti-hypoxic abilities [202].

**Tea** is the most consumed drink in the world after water. The polyphenols present in tea differ according to the type of fermentation performed (green tea infusion 61.86 mg/100 mL; black tea infusion 104.48 mg/100 mL) [177]; green tea is mainly made up of catechins; black tea contains a large amount of tannins. It has been shown that high caffeine consumption can reduce the risk of dementia [203]. In patients with AD, green tea catechin polyphenols have shown neuroprotective properties, such as anti-inflammation, anti-oxidative stress, anti-apoptosis, and the ability to inhibit beta-amyloid protein aggregation. Mancini et al. described encouraging actions of green tea on cognition [204]. Several evidences suggest that green tea polyphenols confer defensive actions on the skin against the acceleration of skin aging induced by UV ultraviolet rays, with anti-melanogenic, anti-wrinkle, antioxidant, and anti-inflammatory effects [205,206]. Systematic consumption of green or black tea can induce the expression of various antioxidant enzymes and hinder oxidative DNA damage [207]. Elmets et al. found that tea extract has a dose-dependent inhibitory action on the erythema caused by UV irradiation [208]. Therefore, the researchers said tea extract could be a natural alternative for photo aging. Added in vivo research on Drosophila has described effects regarding the rise in average lifespan caused by catechins and theaflavins [209].

**Extra virgin olive oil** is characteristic example of polyphenolic-food (55.14 mg/100 g) [177]. A slowdown in aging-related changes has been observed as a consequence of regular olive oil consumption, in cellular, animal, and human models. Prospective studies have revealed how adherence to a MedDiet, a diet characterized by olive oil as the predominant source of fat, is associated with lower mortality, improved health, greater longevity, reduced risk of cardiovascular syndromes, cancer and the incidence of age-related mental deterioration [210]. These benefits have also been associated with the presence of highly bioactive minor molecules in olive oil, including numerous polyphenols such as tyrosol, hydroxytyrosol, oleuropein, aglycone, caffeic acid, and oleocanthal [49,211,212,213].

## 5. Blue Zone, Polyphenolic-Food, and Longevity

Blue zones (BZs) are mysterious areas that continue to fascinate research. They are precise zones in the world where life expectancy is booming—reaching levels far above anywhere else some parts of the world; according to researches, these regions are homes to many nonagenarians. The BZs have been identified in 2005 in an article in the National Geographic by respected journalist and author Dan Buettner [214], they got their name because, when Buettner was researching these areas, he used blue circles around them on the world map. Although Buettner only talks about the five regions in his book, they may not be the only areas in the world that can be classified as BZs.

**Figure 3:**
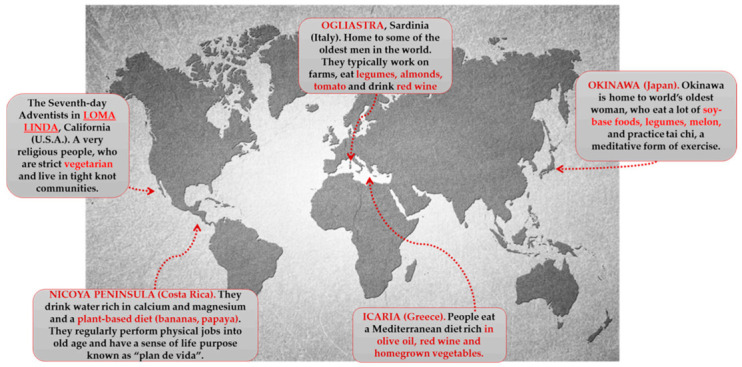
Blue zones of the world.

However, currently, the BZs are (Figure 3):

Characteristic polyphenolic-foods of the Blue zones diets are shown.

Ogliastra (sub region on the Mediterranean island of Sardinia). Located off the coast of Italy, Ogliastra is home to the world’s longest-lived men. This community of shepherds walks five mountainous miles a day or more. This natural movement provides all the helpful cardiovascular benefits. The classic Sardinian diet is plant based, consisting of whole-grain bread, beans, garden vegetables, and fruits. Meat is largely reserved for singular occasions. Sardinians drink wine moderately; Cannonau wine has two or three times the level of flavonoids as other wines [215,216,217].

Island of Okinawa (Japan). Home to the world’s longest-lived women, these South Pacific islands offer many secrets to longevity. The Okinawa tradition of forming a moai provides confident social networks. They meet every day to drink sake and gossip. Okinawans also attribute their longevity to the old Confucian mantra said before meals “Hara Hachi Bu,” which reminds them to stop eating when 80% full, so they do not overeat. They hold a strong sense of purpose in their family. One centenarian described the feeling of holding her great grandchild as “Jumping into heaven” [214,215].

Loma Linda (California). This Seventh Day Adventist religious community outlives the average American by a decade. Taking their diet directly from the Bible, they consume a vegan diet of leafy greens, nuts, and legumes. They recognize the Sabbath and downshift for 24 h every week. The range of diets consumed by Adventists is broad and distinct from the characteristic Western diets [214,218]

Peninsula of Nicoya (Costa Rica). Nicoyans are more than twice as likely as Americans to reach a healthy age of 90 years. Faith and family play a strong role in Nicoyan culture. So does *plan de vida*, or reason to live, which helps Nicoyan elders maintain an optimistic outlook and active lifestyle. Nicoyans eat little to no processed foods but plenty of antioxidant-rich tropical fruit [215,216,219].

Island of Ikaria (Greece). People on this tiny Aegean island live 8 years longer than Americans do. They experience 20% less cancer, half the rate of heart disease, and almost no dementia. Ikarians eat a variant of the MedDiet, with lots of vegetables and fruits, whole grains, beans, potatoes, and olive oil. Ikarians also downshift with a midafternoon break. People who sleep regularly have up to 35% lower chances of dying from heart syndrome [215].

A mix of things makes these places so unique. Genetics can account for a modification of up to 30% in life expectancy amongst humans, but that does not explain the vast dissimilarity in residents of these five zones compared to the rest of the world [220,221]

Modifiable factors, such as physical activity, diet, smoking cessation, and mid-day naps, might depict the “secrets” of the long-life; these findings propose that the interaction of environmental, behavioral, together with clinical characteristics may regulate longevity. This concept must be further studied in order to understand how these elements relate and which are the most significant in shaping prolonged life [214,215,222]. Their longevity is held to be largely due to lifestyle, principally their traditional diet, which is low in calories but rich in nutrients, mainly phytonutrients (polyphenols) [223,224]. Data on centenarians from Southern Italy reinforce these findings [202,225].

Most of the people in the BZs practice fasting of some type, whether it is for religious reasons or intermittent fasting while working during the day. Calorie restriction (CR), not associated with malnutrition, is definitely the best characterized non-genetic intervention that enhances maximum lifespan and improves health span, preventing or retarding the onset of pathophysiological changes in different species [226]. CR slows the aging and enhances the lifespans of fish, flies, mammals (mice and rats), and spiders [227]. CR reduces the oxidative load, which decreases the ROS synthesis in mitochondria. The decrease in the ROS synthesis substantially decreases the amount of oxidized proteins, lipids, and altered mitochondrial DNA. It is supposed that CR and the ingesting of food rich in antioxidants can significantly extend the life span of persons [5,228]. Researchers believe that consuming fewer calories may be contributing to the long life expectancy in some of these blue regions. According to a study, reducing your calorie intake by even 30% can considerably increase life expectancy [229].

Numerous hypotheses have been proposed to understand the cause of the increase in these long-lived populations; the main explanations were based mainly on genetic and environmental factors, on lifestyle, on work activities, and also on social life. Certainly, among the factors related to lifestyle, eating habits play a predominant role: it is now established that the possibility of some individuals to reach an advanced age, managing not to contract most of the chronic non-communicable diseases, is connected at least in part from adopting a healthy diet.

Various nutritional protocols share the merit of being architects of humanity’s longevity. Among these, the MedDiet is considered one of the most capable eating styles to defend against age-related diseases, extending survival. The MedDiet, the traditional dietary model of men who inhabit the lands bathed by the Mediterranean Sea, is characterized by a high intake of olive oil, vegetables, fruit, a controlled consumption of fish, whole grains, and red wine [230], and from a reduced intake of sweets, meat, and dairy products. Olive oil, red wine, nuts, fruits, legumes, and vegetables, key components of the MedDiet, are all polyphenol-rich foods. The much-appreciated MedDiet is well-known for its antioxidant and anti-inflammatory effects. The cardio defensive action of this dietary pattern has been attributed, in part, to the high quantity of antioxidant components such as the phenolic molecules [231,232,233,234,235]. Using the Phenol-Explorer database [177], Godos et al. estimated that an Italian study population had a mean intake of approximately 660 mg of polyphenols per day, obtained from nuts, tea, apples, coffee, cherries, citrus fruits, vegetables, chocolate, and red wine, all regular constituents of the MedDiet, included in the list of the Intangible Cultural Heritage of Humanity by the United Nations Educational, Scientific and Cultural Organization (UNESCO) [236].

A 10-year longitudinal research (HALE) carried out in ten European countries and involving elderly subjects with and without chronic syndromes showed an important association between MedDiet and lifespan [237]. A greater MedDiet adherence has lately been associated with a lower incidence of cardiovascular syndrome and mortality in the UK, suggesting that it promotes healthy aging also in non-Mediterranean countries [238].

Crous-Bou et al. observed that one-third of AD cases are associated to adaptive risk factors, which show the probability for lifestyle interventions such as exercise or MedDiet adherence [239]. Numerous epidemiological studies highlight the potent effects of MedDiet on cognitive impairment and on the risk of AD [240]. Therefore, precautionary plans, such as nutritional regimes, to reduce the role of modifiable risk factors in AD have become indispensable. In this context, one study pointed out that consuming a MedDiet consistently increased levels of cognitive function in older men and women over a period of eleven years [241]. Furthermore, in a population of 1410 Bordeaux citizens, the MedDiet has been linked to less cognitive impairment, as estimated by the Mini-Mental State Examination [242]. High MedDiet adherence reduced the risk of AD in a study involving 2258 New York City residents [243]. In a research on 110 healthy elderly people, divided into half individuals who received a MedDiet and other half subjects who took MedDiet plus extra virgin olive oil, it was shown that the action on mental function was greater in people who consumed a MedDiet with additional supplementation of extra virgin olive oil [244]. Therefore, the intake of bioactive alimentary molecules, contained in olive oil, seems to possess a neuroprotective capacity and is also capable of enhancing the beneficial effects of MedDiet [245].

Cao et al. describe how the consumption of a MedDiet reduces the risk of dementia [246]. In elderly subjects, living in a Polish rural community, greater adherence to MedDiet led to better scores on numerous cognitive function tests [247]. This finding is significant and may have consequences for health policy and practice, as it demonstrates that when the MedDiet was adopted, benefits also manifested in non-Mediterranean populations. Finally, in elderly individuals registered in the Australian Diabetes, Obesity, and Lifestyle Study, it was shown that cognitive function was negatively influenced by Western dietary models, compared to the intake of a plant-based diet [248]. Ideally, an association between the dietary patterns of the elderly population of all five BZs documented so far could help understand the role of nutrition in slowing aging.

The research of the older population of the Nicoya peninsula, linked to those of Ogliastra, proposes a plant-based diet, integrated with a suitable intake of animal proteins, the ideal strategy for maintaining an optimal state of health. [219].

Although these people of BZs are not strict vegetarians, (excluding the Seventh Day Adventists), they limit meat consumption to a maximum of almost five times a month. Their diet is at least 90% plant-based. The BZs diet consists of proteins such as fish instead of meat. High levels of red meat are related to inflammation. Fish is an exceptional source of omega-3 fats, which are good for brain and heart health, as well as for decreasing inflammation [220,249,250]. The BZs diet consists of whole grains, which are rich in fiber; vegetables, another great source of fiber; legumes such as chickpeas, beans, and peas, which are rich sources of protein and fiber; and colorful fruit and vegetables, consuming a diet that is rich in plant phytochemicals such as berries and grapes [220,221,251,252]

There is a deep interest in the Japanese diet on the part of the scientific world to understand, even if partially, the reason for the favorable state of health and longevity of the Japanese people. As an example of an Asian diet, the Okinawan diet has proved very interesting as the inhabitants of the Japanese islands of Ryukyu (the principal island of Okinawa) are some of the healthiest and longest-lived people in the world [253]. The Okinawan diet is characterized by a low calorie and fatty acid intake, a high intake of vegetables and soy products, and a moderate to high intake of fish and sea vegetables [224]. Le Couteur et al. called bitter melon, Okinawan tofu, seaweed and turmeric as features of the Okinawan food [253]. The associations between MedDiet, as a European lifestyle, and the Okinawan diet, as part of an Asian lifestyle, are a high consumption of antioxidants contained in fruits and vegetables, modest to high intake of fish, and checking on healthy fats that are abundant in omega-3 fatty acids and lower in saturated lipids [224,225,254].

Typical foods of the Okinawan diet that are beneficial to health are soy products such as turmeric (curcumin), tofu (spermidine), seaweed (astaxanthin) and seafood [255]. The Asian/Okinawan diet and the MedDiet have some characteristics in common. Resveratrol is associated with the MedDiet, and there is a high concentration of it in the Japanese knotweed, a spring vegetable from East Asia. Therefore, in the two types of diets, there may be an overlap of health promoting bioactive complexes; astaxanthin can also be contained in seafood (for example, shrimp), but is also part of the intake in MedDiet. Spermidine is contained in both diets as it is found in soy and its derivatives, red wine, as well as vegetables and fresh fruit, which are indispensable for MedDiet and Asian/Okinawan diets [225].

Unlike BZ diets, the Western diet, a typical diet of developed countries such as the United States, could have harmful consequences for health due to the high intake of unhealthy fatty acids (saturated), sweets, red meat, and food products, highly transformed [225,256,257].

## 6. Conclusions

**Figure 4:**
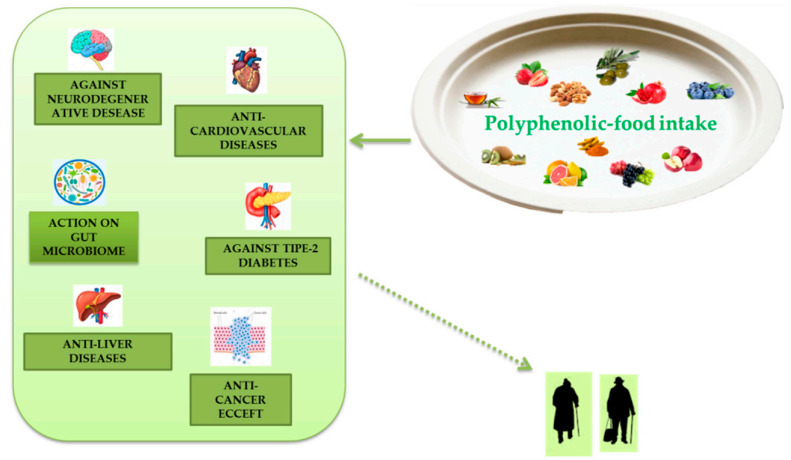
Polyphenolic-food can be useful to combat metabolic and degenerative diseases associated to aging.

Extensive research has demonstrated numerous anti-aging properties of polyphenols, both in cellular, animal, and human models and their ability to slow various diseases associated with aging (Figure 4). Polyphenols are present in plant-based foods and beverages, and the inclusion of polyphenolic-foods in the diet is consistent with the advice to eat five or more servings of fruit and vegetables a day. The advantages due to polyphenolic-food consumption can be caused by their antioxidant properties, direct or indirect. These effects can be further increased by the ability of these molecules to improve endogenous antioxidant systems with consequent reduction of oxidative stress, a phenomenon related to aging in tissues. A useful relationship between leukocyte telomere dimension and MedDiet adherence has been described, and a study directly estimating the properties of polyphenol intake on telomere size and their actions on telomerase activity certainly deserves a review further examination. These phytochemicals can induce epigenetic changes. Their influence on proteostasis is apparently age- and tissue-specific. These phytochemicals also appear to display beneficial effects on stem cell function and have been documented as significant vital promoters of tissue regeneration both in vitro and in vivo. Inflammation is another important mark of polyphenols which can be described by the anti-inflammatory effect.

Several studies report how MedDiet and other Blue zones diets are related to improvements in mental degeneration in aging that may be related to the healthful effects of polyphenolic food consumed in these diets.

Anyway, the recognition that the useful actions of polyphenols are used on practically all the hallmarks of aging proposed both in cellular models and in organisms could help us to comprehend the molecular basis of health development, reduction of the risk of syndromes linked to aging, and augmented longevity that has been linked to the intake of BZs diets, rich in polyphenolic foods. Future research on this interesting topic is clearly justified.
